# Effect of Electroacupuncture on Shoulder Subluxation in Poststroke Patients with Hemiplegic Shoulder Pain: A Sham-Controlled Study Using Multidimensional Musculoskeletal Ultrasound Assessment

**DOI:** 10.1155/2021/5329881

**Published:** 2021-11-19

**Authors:** Minghong Sui, Naifu Jiang, Luhui Yan, Jiaqing Liu, Bin Luo, Chenxi Zhang, Tiebin Yan, Yun Xiang, Guanglin Li

**Affiliations:** ^1^Department of Rehabilitation Medicine, Huazhong University of Science and Technology Union Shenzhen Hospital (Shenzhen Nanshan People's Hospital), Shenzhen 518052, China; ^2^CAS Key Laboratory of Human-Machine Intelligence-Synergy Systems, Shenzhen Institute of Advanced Technology (SIAT), Chinese Academy of Sciences (CAS) and the SIAT Branch, Shenzhen Institute of Artificial Intelligence and Robotics for Society, Shenzhen 518055, China; ^3^Guangdong-Hong Kong-Macao Joint Laboratory of Human-Machine Intelligence-Synergy Systems, Shenzhen 518055, China; ^4^Department of Rehabilitation Medicine, Sun Yat-sen Memorial Hospital, Sun Yat-sen University, Guangzhou 510120, China; ^5^Department of Sports Rehabilitation, School of Sports Medicine and Health, Chengdu Sport University, Chengdu 610041, China

## Abstract

**Objective:**

This study aimed to use multidimensional musculoskeletal ultrasound imaging technique to investigate the effect of electroacupuncture (EA) on shoulder subluxation in poststroke patients with hemiplegic shoulder pain.

**Methods:**

In this prospective single-blind, randomized, sham-controlled study, thirty-four patients with shoulder subluxation and hemiplegic shoulder pain were recruited and randomly assigned into the EA group or the sham EA (SEA) group. In the EA group, EA was applied to the Jian yu (LI15), Bi nao (LI14), Jian zhen (SI9), and Jian liao (TE14) acupoints. In the SEA group, the EA was applied 15 mm away from the Lou gu (SP7), Di ji (SP8), Jiao xin (KI8), and Zhu bin (KI9) acupoints. Both groups underwent treatment 30 minutes/day, five days a week, for two weeks using dense waves with a frequency of 2/100 Hz. A Visual Analogue Scale (VAS) was used to evaluate the effectiveness of treatment in reducing shoulder pain. Musculoskeletal ultrasound was used to evaluate the changes of measures of shoulder subluxation in multidimensions (i.e., the acromiohumeral distance, AHD; acromion-greater tuberosity, AGT; and acromion-lesser tuberosity, ALT). Both the within- and between-groups treatment effects were assessed.

**Results:**

The pain intensity measured by VAS and shoulder subluxation measured by musculoskeletal ultrasound (i.e., AHD, AGT, and ALT) showed significant (*p* < 0.05) within-group difference in both groups. The between-group difference appeared in the pain intensity (*p* < 0.05), while it disappeared in the three measures of shoulder subluxation (*p* > 0.05).

**Conclusions:**

Using VAS for measuring pain intensity and multidimensional musculoskeletal ultrasound imaging technique for measuring shoulder subluxation, this study finds that the hemiplegic shoulder pain can be improved significantly by the EA while the shoulder subluxation cannot be. Our findings further reveal the analgesic mechanism of EA on hemiplegic shoulder pain following stroke.

## 1. Introduction

Stroke is a common neurological disease noted for its high morbidity, residual disability, and mortality rate [1, 2]. It places a significant burden on families and society [3]. Subluxation of the shoulder joint is a common complication of stroke [4]. Its incidence is as high as 81%, and it usually occurs within three months of the onset of stroke [4]. Subluxation of the shoulder joint is a possible cause for hemiplegic shoulder pain, which seriously affect the recovery of upper limb function [5]. This, in turn, aggravates the economic burden of stroke on family and society and brings immense pain to hemiplegic stroke survivors [6].

Commonly used treatments for shoulder subluxation include electrical stimulation, strapping, and/or orthosis [7–9]. However, there is insufficient evidence to confirm that these treatments definitely reduce shoulder subluxation and promote functional recovery of the upper limbs [7–9]. Thus, it is necessary to find other effective methods for treating shoulder subluxation. Electroacupuncture (EA), as a complementary and alternative therapy, is a form of acupuncture which involves a small pulsed electric current delivered to single or pairs of acupuncture needles [10]. It combines electrical stimulation with traditional acupuncture. Studies have found that EA has significant analgesic effects [11], improves limb muscle strength [12], and improves limb function [13]. Research also indicates that EA applied to the scapula and arm may effectively treat shoulder subluxation [14]. Thus, EA is a potential effective therapy to improve the shoulder subluxation in the stroke patients with hemiplegic shoulder pain.

The analgesic mechanism of EA is still being investigating. It has been studied at peripheral, spinal, and supraspinal levels which involve a series of bioactive molecules [15]. These molecules can be regulated and modified by EA and the relevant nociceptive neurons can also be impacted [16]. It is indicated that EA can relieve shoulder pain effectively through neural-immune-endocrine interactions [16, 17]. Because many clinical factors including physiological and psychological factors are highly correlated to the pain symptom [17, 18], it is also significant to investigate the analgesic mechanism of EA by measuring these clinical factors. Taking these factors into consideration, the relationship between the occurrence of shoulder subluxation and the development of hemiplegic shoulder pain is not consistent in the literature of stroke [19, 20]. By analyzing the alteration of hemiplegic shoulder pain and shoulder subluxation following EA, it will help to explore the relationship between the shoulder subluxation and the EA as well as the hemiplegic shoulder pain.

In order to assess the alteration of shoulder subluxation following EA and explore the possible analgesic mechanism of EA, an appropriate evaluation tool should be applied. The musculoskeletal ultrasound imaging is a noninvasive objective tool to diagnose the shoulder subluxation [21, 22]. Compared to other radiological examinations, it shows advantages including excellent visualization of shoulder structure, lack of ionizing radiation, dynamic assessment, uncomplicated operation, and cost-effectiveness [23]. By measuring the distance between two osseous landmarks, including acromiohumeral distance (AHD) [24], acromion-greater tuberosity (AGT) [22], and acromion-lesser tuberosity (ALT) [24, 25], musculoskeletal ultrasonography can accurately compute the degree of subluxation in multidimensions [25]. This enables objective evaluation of treatment effect and exploration of its mechanism.

Thus, this study aimed to use multidimensional musculoskeletal ultrasound imaging technique to objectively evaluate the effect of electroacupuncture on shoulder subluxation in stroke patients. The findings may help to further understand the treatment mechanism of EA in stroke.

## 2. Materials and Methods

### 2.1. Participants

Patients hospitalized with shoulder subluxation and hemiplegic shoulder pain after stroke were recruited from the Huazhong University of Science and Technology Union Shenzhen Hospital (China) from October 2018 to September 2019. The sample size in this study was computed according to previous literature on EA treatment for hemiplegic shoulder pain [26]. By using GPower 3.1.9.2 software, the sample size was estimated to be 34 in this study, while the effect size is 1.11, *α* is 0.05 with a power of 80%, and patient shedding rate is 20%. This study's protocol was approved by the institution's ethics committee (IRB no. 032502). It was also registered with the Chinese Clinical Trial Registry (no. ChiCTR2000029051). All patients (and/or their families) provided informed written consent prior to study participation.

To be included in the study, patients were required to meet the diagnostic criteria for stroke as defined by the Chinese Guidelines for the Prevention and Treatment of Cerebrovascular Diseases [27], be diagnosed using computerized tomography (CT) or magnetic resonance imaging (MRI), and meet the diagnostic criteria for fingerbreadth palpation of shoulder subluxation [28, 29]. This third criterion required that the degree of subluxation be more than half a fingerbreadth gap [28]. Other inclusion criteria were aged 30–75 years; first stroke or previous stroke without sequelae; subluxation that appeared within one year of the stroke; limb dysfunction on only one side of the body; stable vital signs; no severe heart, lung, liver, or kidney dysfunction; no coagulation dysfunction; and visual analogue scale (VAS) pain score ≥4 points. The exclusion criteria were any history of rotator cuff injury; periarthritis, shoulder surgery, or shoulder trauma; malignant tumor; quadriplegia; severe speech or cognitive dysfunction; mental illness; pain caused by cancer, menopause, or fracture; and poststroke depression. We also excluded individuals with severe dizziness or a pacemaker.

### 2.2. Experimental Design

The study is a prospective single-blind, randomized, sham-controlled study. The recruited patients were assigned to EA group or sham EA (SEA) group randomly. All patients enrolled in the study were grouped using a simple randomization method and table of random numbers. Patients were randomly assigned envelopes with randomization number by physicians not participating in the study. If the selected numbers were even, the patients were assigned to the EA group; if the selected numbers were odd, the patients were assigned to the SEA group; if the two groups were not balanced, a random number table was further used to evenly distribute them (ratio: 1 : 1).

In addition to EA treatment, both groups of patients also received conventional drug and rehabilitation treatment. Conventional drug treatment followed the Chinese Cerebrovascular Disease Prevention and Treatment Guidelines [27]. In order to avoid the bias between EA group and SEA group, we recruited the patients who received the identical series of conventional rehabilitation treatments including good limb positioning, passive shoulder movement, active shoulder strapping, rood therapy, weight training of the affected limb, and electrical stimulation therapy. All recruited patients underwent conventional rehabilitation treatments once a day, five days a week, for two weeks. If the type of the patient's rehabilitation treatment changed, this patient would be excluded from this study. By this means, the baseline conventional rehabilitation treatments of both groups can be consistent.

The details of all conventional rehabilitation treatments are as follows:

- Good limb positioning
- Lying on the uninjured side: Shoulder joint was bent as much as possible; then the joints of the elbow, wrist, and fingers were stretched, respectively, on a cushion.
- Lying on the affected side: put a cushion to support the back, the body torso was bent slightly backward so that the shoulder of the affected side was extended, the forearm and fingers of the affected side were naturally stretched, and the palm of the hand was upward.
- Lateral decubitus position: A cushion was placed behind the shoulder joint on the affected side so as to make the shoulder blades maintain extended forward, and upper extremity was naturally stretched, and the palm of the hand was upward.
- Passive shoulder movement
- Making gentle, slow passive movement for joints of the shoulder, elbow, wrist, and fingers of the affected side to avoid excessive strain on the shoulder tissue.
- Active shoulder strapping
- The patient was lying on the uninjured side, the therapist placed one hand in the acromion of the affected side, and one hand passed through the affected side armpit and placed the palm of the hand in the medial lower angle of the scapula. Both hands were used to lift the shoulder and drop and retract the scapula.
- Rood therapy
- The therapist held up the affected side of the upper arm to ensure shoulder abduction, parallel to the long axis of the upper limb repeatedly stimulate the shoulder joint capsule, and told the patient to try to maintain confrontation with the treatment, while stimulating the shoulder muscles such as the deltoid muscle and supraspinatus muscle. Finally, the therapist will be relative to the palms of both hands, gently squeeze the affected shoulder joint.
- Weight training of the affected limb
- The therapist instructed the patients to straighten their upper arm and forearm of the affected side and bend the wrist back after sitting down and then put the palm down on the hard plane on one side of the body. The therapist helped the patient to slowly tilt their upper body to the affected side, and make the shoulder joint of the affected side bear weight of the upper body.
- Electrical stimulation therapy
- The low-frequency neuromuscular electrical stimulation (NMES) was applied to patients. The frequency of stimulation was 1 Hz, while the intensity of stimulation was 20 mA–30 mA depending on the patients' tolerance.

### 2.3. Electroacupuncture (EA) Treatment

In the EA group, EA was applied to the jian yu (LI15), bi nao (LI14), jian zhen (SI9), and jian liao (TE14) acupoints. The positioning and depth were as recommended by Gao Shuzhong in his textbook Acupuncture and Moxibustion therapy [30]. During treatment, the patient was in a side-lying position, and the local skin was disinfected with 75% alcohol. The Hua tuo acupuncture needles were inserted 1–1.5 inches vertically into the skin. The needles were lifted and twisted to produce a feeling of deqi (i.e., sensation of soreness, numbness, distention, or radiating, which is considered to indicate effective needling). The acupuncture was followed by 30 minutes of electroacupuncture performed with a HANS-200A instrument (Suzhou Medical Supplies Ltd., China) using dense waves at 2/100 Hz. Patients underwent treatment once a day, five days a week, for two weeks.

### 2.4. Sham Electroacupuncture (SEA) Treatment

The SEA group received the same treatment as the EA group except for the location of needle insertions—the needles were applied 15 mm from the lou gu (SP7), di ji (SP8), jiao xin (KI8), and zhu bin (KI9) points [31, 32]. Specifically, after disinfection, Hua tuo acupuncture needles 1–1.5 inches long were inserted vertically into the skin of the side-lying participant, to a depth of five millimeters. Following the acupuncture, EA was applied using the same stimulation parameters as for the EA group.

### 2.5. Clinical Outcome Measurements

Outcome measurements were conducted before and after two weeks of treatment. Measures of shoulder subluxation were obtained by the same rehabilitation physician using a uSmart 3300 musculoskeletal ultrasound system (Terason Ultrasound Imaging System Version 5.11.4, frequency 3–17 Hz, USA). Measure of pain intensity was conducted by using Visual Analogue Scale (VAS).

#### 2.5.1. Acromiohumeral Distance (AHD)

**Figure 1:**
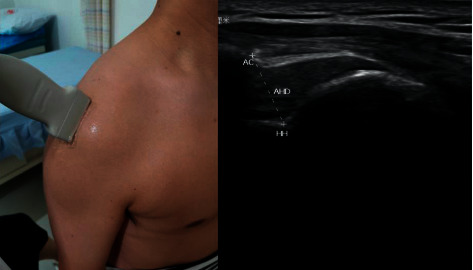
Measurement of acromiohumeral distance (AHD) for shoulder subluxation by using musculoskeletal ultrasound imaging technique. AC indicates acromion; HH indicates humerus head.

The musculoskeletal ultrasound probe was placed at the anterior border of the acromion. When both the acromion and humerus head appeared on the screen, the image was frozen, and the shortest distance between the acromion and humerus head was measured. We calculated the difference in AHD values before and after treatment (Figure 1).

#### 2.5.2. Acromion-Greater Tuberosity (AGT)

**Figure 2:**
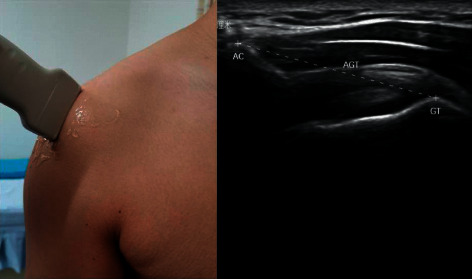
Measurement of acromion-greater tuberosity (AGT) for shoulder subluxation by using musculoskeletal ultrasound imaging technique. AC indicates acromion; GT indicates greater tuberosity.

A musculoskeletal ultrasonic probe was placed on the lateral edge of the acromion and the lateral edge of the long head of the biceps tendon. When the lateral edge of the acromion and the upper edge of the greater tuberosity appeared on the screen at the same time, the image was frozen, and the AGT was measured. We calculated the difference in AGT values before and after treatment (Figure 2).

#### 2.5.3. Acromion-Lesser Tuberosity (ALT)

**Figure 3:**
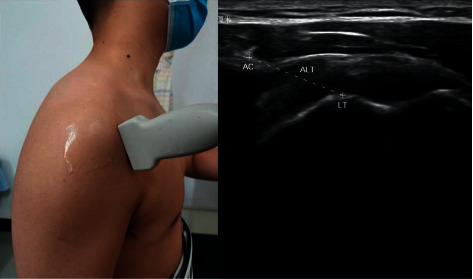
Measurement of acromion-lesser tuberosity (ALT) for shoulder subluxation by using musculoskeletal ultrasound imaging technique. AC indicates acromion; LT indicates lesser tuberosity.

The musculoskeletal ultrasonic probe was placed on the lateral edge of the acromion and the medial edge of the long head of the biceps tendon. When the lateral edge of the acromion and the upper edge of the lesser tuberosity appeared on the screen at the same time, the image was frozen, and the ALT was measured. We calculated the difference in ALT values before and after treatment (Figure 3).

#### 2.5.4. Visual Analogue Scale (VAS) for Shoulder Pain

Shoulder pain was evaluated using a VAS. Specifically, the patient graded their degree of pain on a 10 cm scale with 1 cm marked intervals (where 0 = not painful at all, 10 = unbearable pain, and the interval between represented a gradual increase in pain).

### 2.6. Statistical Analysis

IBM SPSS Statistics for Windows, version 23.0 (IBM Corp., Armonk, NY, USA) was used for statistical analysis. The Shapiro–Wilk test was used to determine whether the data were normally distributed. Descriptive statistics were presented using the mean ± standard deviation (normally distributed data) or median (%25, %75) (nonnormally distributed data) for continuous variables, or numbers for categorical variables. Independent sample *t*-test and *χ*^2^ test were used to compare the baseline characteristics between groups. When data followed normal distribution, paired *t*-test was used for within-group comparison and independent sample *t*-test was used for between-group comparison. Otherwise, Mann–Whitney *U* tests were used to compare nonnormally distributed data. The threshold of statistical significance was set to *p* < 0.05.

## 3. Results

### 3.1. Demographic and Clinical Baseline Characteristics

Two patients in the SEA group dropped out of the study due to being unable to tolerate the pain associated with acupuncture. Thus, 32 individuals (17 in the EA group and 15 in the SEA group) were included in the analyses. Participants included 22 men and 10 women. The mean ages of the EA and SEA groups were 51.00 ± 12.44 years (range = 32–68 years) and 54.40 ± 8.16 years (range = 41–68 years), respectively. The median hemiplegia duration was 64 (range = 14–188) days in the EA group and 65 (range = 12–183) days in the SEA group. There were no statistically significant differences between the EA and SEA groups in gender, age, disease course, or stroke type (Table 1).

### 3.2. Comparison of Pain Intensity

**Figure 4:**
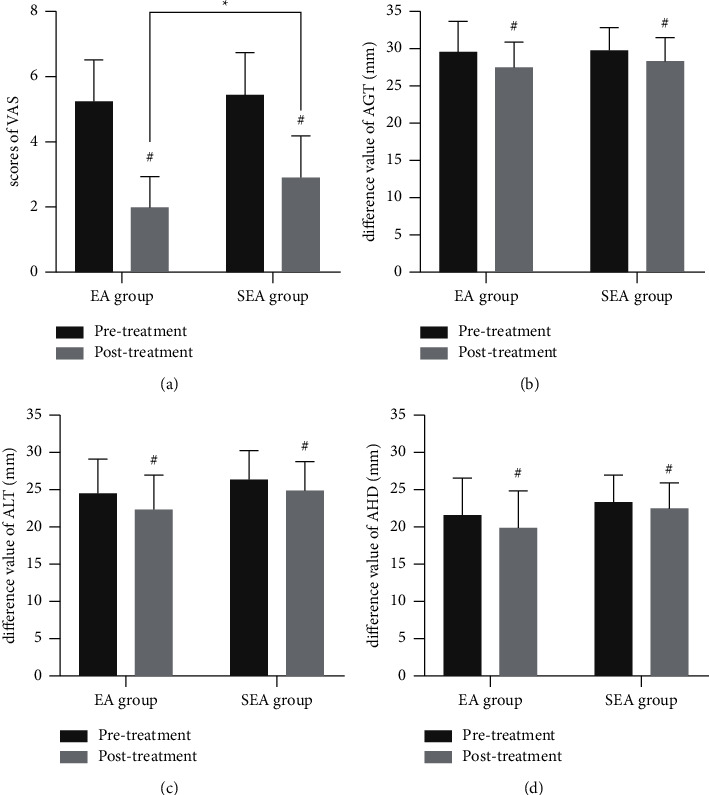
Comparison of VAS scores and measures of shoulder subluxation distances between the two groups before and after treatment. Comparison within groups: ^#^*p* < 0.05. Comparison between groups: ^*∗*^*p* < 0.05.

Before treatment, there were no significant differences in average VAS scores between the two groups. After two weeks of treatment, the average VAS scores of both groups were significantly lower than before treatment (*p* < 0.05). Moreover, the EA group's average VAS score was lower than the SEA's group. There were significant differences in the average VAS scores of both groups (*p* < 0.05) (Table 2 and Figure 4). By using independent *t*-test to compare the change of VAS, it showed a significant difference between EA and SEA groups (change of VAS: 3.29 ± 1.04 vs. 2.53 ± 0.83, *p*=0.03).

### 3.3. Comparison of Ultrasound-Based Measures of Shoulder Subluxation

Before treatment, there were no significant differences in the average AHD, AGT, and ALT values between the two groups. After treatment, the average AHD, AGT, and ALT values were lower in both groups than before the treatment (*p* < 0.05). However, there were no significant between-group differences in the reductions in AHD, AGT, or ALT values (Table 3 and Figure 4). By using independent *t*-test to compare the change of ultrasound-based measures, it showed no significant difference between EA and SEA groups in all measures (change of AHD: 1.74 ± 2.30 vs. 0.87 ± 1.43, *p*=0.21; change of AGT: 2.02 ± 3.34 vs. 1.43 ± 1.49, *p*=0.52; and change of ALT: 1.99 ± 2.72 vs. 1.41 ± 1.26, *p*=0.44).

## 4. Discussion

In this study, we compared EA with SEA so as to provide further clinical evidence for the effectiveness of EA in the treatment of shoulder subluxation as well as hemiplegic shoulder pain following stroke. Findings from the VAS indicated that, combining with conventional drug and rehabilitation therapy, the EA treatment could reduce more shoulder pain than SEA treatment. Specifically, the EA group showed statistically significant improvement in shoulder pain as compared with the SEA group. However, there were no significant between-group differences in changes of measures of shoulder subluxation via musculoskeletal ultrasound examination.

This study found that after two weeks of treatment, the analgesic effect of the EA group was significantly better than that of the SEA group, indicating that EA can effectively relieve shoulder pain. However, it is unclear whether this treatment produces long-term analgesic effects. Electroacupuncture is widely used in the clinical treatment of pain. As a form of acupuncture, EA's analgesic effects have been widely established in various conditions such as inflammatory pain, postoperative pain, and pathological neuralgia [33–35]. Many studies have proved that EA has analgesic effect, but the analgesic mechanism of EA is not completely clear. Studies have shown that EA may promote local blood circulation, accelerate the absorption of local inflammatory substances, nourish nerves, repair damaged tissues, stimulate the brain to release endogenous morphine substances, and improve the pain threshold [36]. Wang et al. [37] used traditional acupuncture in the treatment of poststroke shoulder pain and found that traditional acupuncture may treat poststroke shoulder pain by promoting the release of endorphins, nourishing muscle fiber repair, and breaking the pain-immobilization-pain cycle. A study by Li et al. [38] used EA combined with massage to treat poststroke shoulder pain. Their results suggested that EA combined with massage may reduce abnormal shoulder movement patterns, promote local blood circulation, reduce local tissue adhesion, and have analgesic effects. Multiple studies have also shown that EA may have a significant analgesic effect in the treatment of shoulder subluxation and hemiplegic shoulder pain after stroke [39–41]. Proposed mechanisms include improvements in shoulder joint adhesions, muscle strength improvements, and relief in the pull on the shoulder capsule (and thus shoulder pain) [39–41]. Some researchers discussed the possible mechanisms of acupuncture analgesia from three aspects (nerve, body fluid and enzyme) and found the pain is mainly due to the nerve mediation [10, 42]. These papers mainly verify the analgesic mechanisms of electroacupuncture from the aspect of the neuromuscular system.

Although previous studies showed that EA treatment may minimize shoulder joint subluxation [39, 40], there is no concrete evidence to support this assertion. In this study, musculoskeletal ultrasound was used to evaluate the effect of EA treatment on shoulder subluxation in stroke patients with hemiplegic shoulder pain. The findings show that there is no significant difference in reducing the distance of shoulder joint subluxation between EA and SEA groups. It indicates the decrease of pain intensity can be due to different modulating mechanisms and lead to increased function without a need of structural changes, as this has widely been demonstrated in many other areas of research [42, 43]. The EA can sustain local muscle relaxation and contraction. In this study, the EA point of the shoulder was just around the deltoid and the supraspinatus muscle. This was chosen to stimulate the corresponding acupoints and increase muscle strength, as well as potentially strengthen the traction and contraction of the deltoid and supraspinatus muscles on the humeral head. Watson [44] also demonstrated that electrical stimulation may accelerate the recovery of related muscle functions, while Zhou et al. [45] determined that electrical stimulation may improve the upper limb function.

The results of this study showed that there was no correlation between hemiplegic shoulder pain and shoulder subluxation. This finding was also displayed in some previous studies [43, 46]; there was no clear causal relationship between pain and structural changes. Hemiplegic shoulder pain is a complex phenomenon whose pathophysiological mechanism is not fully clear [47]. Shoulder subluxation is considered to be the most significant risk factor for shoulder pain [4]. It is caused by the dislocation of the humeral head from the glenoid, and its clinical manifestation is usually marked by a depression between the acromion and the humeral head [5]. Common causes of shoulder subluxation are reduced strength of the muscles around the shoulder (especially the medial and lateral parts of the deltoid and supraspinatus muscles) [48], the shoulder capsule and surrounding soft tissue being pulled and relaxed under the effect of gravity, uneven tension, and changes in the brachial-lump rhythm, poor limb placement, and improper stretching of the upper limbs [49, 50]. Since pain sensors in the shoulder joint capsule are more susceptible to damage in shoulder subluxation, most patients experience shoulder pain [51]. Nonetheless, currently, more and more researchers believe that the occurrence of hemiplegic shoulder pain is multifactor. It can be classified according to three aspects: impaired motor control (altered muscle tone) [51], soft tissue injury (shoulder subluxation [20, 52], biceps longhead tendinopathy [52], supraspinatus tendinopathy [19]), and changes in peripheral and central nervous activity [46, 47]. The relationship between the shoulder subluxation and pain needs a larger sample size study.

This study still has some limitations. Unlike the majority of other studies, ours only included Chinese stroke survivors. Participants' conventional treatment programs could not be completely standardized, but every effort was made to reduce heterogeneity between patients. Use of musculoskeletal ultrasonic evaluation could have reduced the error, including obtaining as many ultrasonic results as possible to take the average value of multiple evaluations. Finally, future studies should use larger sample sizes and contain longer follow-up periods. We plan to address these limitations and further clarify the therapeutic effects of EA on shoulder subluxation and hemiplegic shoulder pain following stroke in future work.

## 5. Conclusions

Using the VAS for measuring pain intensity and multidimensional musculoskeletal ultrasound imaging technique for measuring shoulder subluxation, this study finds that the hemiplegic shoulder pain can be improved significantly by the EA while the shoulder subluxation cannot be. Our findings further reveal the analgesic mechanism of EA on hemiplegic shoulder pain following stroke.

## Figures and Tables

**Table 1:** Participants' demographic characteristics.

Group	Gender		Age	Course of disease	Stroke type	
	Male	Female	Years, mean ± SD	Days, median (25%, 75%)	Hemorrhage	Infarction
EA group	12	5	51.00 ± 12.44	33.00 (23.00, 114.00)	7	10
SEA group	10	5	54.40 ± 8.16	44.00 (25.00, 112.00)	5	10
*P* value	0.811^*∗*^		0.363^#^	0.961^#^	0.647^*∗*^	

^*∗*^ indicates *χ*^2^ test; # indicates independent *t*-test. SD indicates standard deviation.

**Table 2:** Scores of VAS before and after treatment.

Observation indicator	Group	Pretreatment	Posttreatment
VAS	EA group	5.29 ± 1.26	2.00 ± 0.94^*∗*^●
	SEA group	5.47 ± 1.30	2.93 ± 1.28^*∗*^

^*∗*^ indicates a significant improvement compared with before treatment, *p* < 0.05. ● indicates a significant between-group difference after 2 weeks of treatment, *p* < 0.05. Data are expressed as mean ± standard deviation (SD).

**Table 3:** Measures of shoulder subluxation distances before and after treatment.

Observation indicators	Group	Pretreatment	Posttreatment
AHD (millimeter)	EA group	21.74 ± 4.71	20.00 ± 4.88^*∗*^
	SEA group	23.36 ± 3.58	22.49 ± 3.46^*∗*^

AGT (millimeter)	EA group	29.71 ± 4.08	27.69 ± 3.27^*∗*^
	SEA group	29.98 ± 2.94	28.55 ± 3.12^*∗*^

ALT (millimeter)	EA group	24.62 ± 4.60	22.62 ± 4.44^*∗*^
	SEA group	26.52 ± 3.86	25.10 ± 3.96^*∗*^

^*∗*^ indicates a significant difference as compared with before treatment by using paired *t*-test, *p* < 0.05. Data are expressed as mean ± standard deviation (SD).

## Data Availability

The data used to support the findings of this study are available from Dr. Minghong Sui (e-mail: meekoo@163.com) upon request.
